# Informer-based DDoS attack detection method for the power Internet of Things

**DOI:** 10.1371/journal.pone.0322329

**Published:** 2025-05-29

**Authors:** Wei Cui, Xiao Liao, Yang Yang, Shiying Feng, Mingyan Song

**Affiliations:** 1 State Grid Information and Telecommunication Group Co., Ltd., Beijing, China; 2 School of Automation Science and Engineering, Xi’an Jiaotong University, Xi’an, China; Cardiff Metropolitan University - Llandaff Campus: Cardiff Metropolitan University, UNITED KINGDOM OF GREAT BRITAIN AND NORTHERN IRELAND

## Abstract

With the rapid development of smart grids, power grid systems are becoming increasingly complex, posing significant challenges to their security. Traditional network intrusion detection systems often rely on manually engineered features, which are not only resource-intensive but also struggle to handle the diverse range of attack types. This paper aims to address these challenges by proposing an automated DDoS attack detection algorithm using the Informer model. We introduce a windowing technique to segment network traffic into manageable samples, which are then input into the Informer for feature extraction and classification. This model captures both the temporal dependencies and global attention information in the traffic data. Experimental results on the CICIDS-2018 dataset demonstrate the effectiveness of our approach, showing significant improvements in detection accuracy and efficiency. Our findings suggest that the proposed method offers a promising solution for real-time intrusion detection in complex power grid environments.

## 1 Introduction

The rise of the Internet of Things (IoT) marks the beginning of a new era defined by remarkable advancements. In this framework, everyday objects are embedded with sensors, software, and connectivity, allowing them to collect, share, and respond to data [1]. This transformative change has influenced many areas of modern life, including smart homes, wearable technology, industrial automation, and healthcare. One of the most groundbreaking applications of IoT technology is found in power systems, leading to the development of the Power Internet of Things (Power IoT) [2].

Power IoT embodies the fusion of conventional power infrastructure with cutting-edge IoT capabilities. It encompasses a wide range of devices and sensors integrated into the electrical grid, including smart meters, distribution management systems, renewable energy sources, and grid-edge controllers [3]. Together, these devices create a sophisticated network that coordinates the generation, distribution, and consumption of electrical energy in ways that were previously unimaginable. Power IoT holds the potential to elevate the efficiency, dependability, and sustainability of power grids [4], ushering in a new era of intelligent energy management and grid optimization.

The integration of IoT technology into power systems offers unprecedented benefits but also ushers in a new set of challenges [5], with the foremost among them being the heightened vulnerability to cyber threats. Distributed Denial of Service (DDoS) attacks, a particularly insidious form of cyber assault, have the potential to disrupt Power IoT infrastructures [6], imperiling the availability of electrical energy and compromising grid stability. And to counter the ever-growing threat of Distributed Denial of Service (DDoS) attacks in the realm of Power IoT, traditional detection methods have predominantly relied on rule-based approaches and anomaly detection techniques. Rule-based methods often entail the creation of predefined rules to identify known attack patterns. While effective to some extent, these rules are inflexible and struggle to adapt to the evolving nature of DDoS attacks [7], frequently resulting in high false-positive rates and limited coverage against novel attack vectors. Conversely, anomaly detection methods leverage statistical models to identify deviations from expected network behavior. While they are capable of detecting previously unknown attacks, they tend to generate numerous false alarms due to their sensitivity to normal network fluctuations. Additionally, these methods often face challenges in capturing the intricate temporal and spatial dependencies inherent in DDoS attacks, rendering them less suitable for the complex and dynamic Power IoT environments.

While traditional DDoS detection methods have limitations, deep learning offers a promising alternative by capturing complex patterns and autonomously recognizing subtle DDoS attack signatures. Leveraging the vast network data generated in Power IoT environments, deep learning approaches aim to improve detection accuracy and adaptability.

However, detecting DDoS attacks in Power IoT faces significant challenges. Current methods struggle to generalize to the diverse and dynamic traffic patterns of these networks, while the resource constraints of IoT devices demand lightweight, efficient models. These challenges highlight the necessity of developing tailored solutions, such as the approach proposed in this study.

The primary motivations for this study stem from the increasing sophistication of DDoS attacks and the limitations of existing detection methods. Traditional approaches struggle to process large-scale, dynamic network traffic efficiently, highlighting the need for advanced models capable of handling these challenges. To address these issues, this paper makes the following contributions:

- We introduce a framework that integrates the Informer model into DDoS attack detection, leveraging its strengths in processing time-series data with sparse self-attention mechanisms.
- Extensive experiments conducted on the CICIDS-2018 dataset demonstrate the superiority of the proposed approach in terms of detection accuracy and computational efficiency.

## 2 Related works

The primary objective of DDoS attack detection is to identify behaviors attempting to breach the security boundaries of protected systems through the network. Successful DDoS attack detection empowers network administrators to promptly recognize and respond to malicious traffic, thereby establishing a robust defense at the network security level. This, in turn, ensures the stable provision of network services and the continuous normal operation of business. DDoS detection, as a pivotal element in DDoS defense efforts, is highly esteemed by scholars both domestically and internationally. Presently, common DDoS detection methods can be broadly classified into three categories: statistical detection methods, traditional machine learning-based detection methods, and deep learning-based detection methods.

Statistical-based methods require analyzing the inherent statistical characteristics of network traffic. By examining fundamental statistical information like mean, variance, and frequency, the detection system identifies anomalous traffic indicative of potential DDoS attacks. A common statistical approach for DDoS detection involves investigating entropy anomalies in traffic data packet fields. For instance, during an attack, the distribution of destination IP addresses decreases while the distribution of source IP addresses increases [8]. Statistical modeling involves setting appropriate thresholds and classifying new traffic data to determine if it constitutes attack traffic [9]. However, obtaining representative samples of normal traffic across diverse network environments is challenging and often relies on manually selected thresholds. Establishing suitable detection thresholds based on network environments increases complexity, requiring higher professional expertise and influenced by human factors affecting detection accuracy. Additionally, as the Internet scales and complexity grows, monitoring traffic attributes struggles to provide sufficient information for effective detection models. As a result, these methods are gradually becoming less prominent.

Machine learning technology learns from data features to create models, aiding decision-making. It’s widely used in DDoS detection to train classifiers distinguishing attack from normal traffic. Traditional models like SVM and decision trees excel in this area. For example, Dong *et al*. [10] showed SVMRBM performed best, combining RBM tech. However, traditional machine learning struggles with complex DDoS landscapes and relies heavily on feature engineering, with human factors impacting accuracy. Deep learning is gaining traction for intrusion detection, potentially replacing traditional methods [11]. Saheed [12,13] *et al*. proposed the IoT-Defender framework, which combines MGA and LSTM for intrusion detection, achieving 99.41% accuracy on the BoT-IoT dataset. They also introduced GA-mADAM-IIoT, an optimized LSTM model with a genetic algorithm and attention mechanism, achieving 99.98% accuracy on the SWaT dataset and 99.87% on the WADI dataset.

With the rise of ChatGPT and advancements in natural language processing, the Transformer architecture [14], as proposed in the paper “Attention is all you need," has garnered widespread recognition since 2017. Its adaptability from natural language processing tasks to prediction and classification challenges in sequential and non-sequential contexts has shown promising outcomes. Moreover, researchers are increasingly integrating Transformer structures and Attention mechanisms into their work, particularly in fields such as DDoS attack detection, where models like the MTNN have demonstrated significant improvements in accuracy and performance [15].

In long-term prediction and classification tasks, Zhou *et al*. [16] introduce the Informer model as an alternative to Transformer, aiming to address its limitations. While Transformer excels in expressing long-range dependencies, it faces challenges in Long Sequence Time Forecasting (LSTF) tasks due to its high time complexity and memory usage per layer, along with prolonged prediction times for longer sequences. Informer proposes three improvements: ProbSparse self-attention, self-attention distillation, and a generative decoder, reducing algorithm complexity and generation time significantly. In the realm of DDoS attack detection, where real-time processing and swift response are critical, this study evaluates the performance of Informer.

Despite progress in DDoS detection, existing methods still face challenges in balancing detection accuracy and computational efficiency, particularly in handling large-scale, complex network traffic. These limitations highlight the need for a more efficient and scalable solution.To address this, we introduce a framework that integrates the Informer model into DDoS attack detection, utilizing its sparse self-attention mechanism for effective time-series processing. Experiments on the CICIDS-2018 dataset demonstrate the model’s superior performance in both accuracy and efficiency.

## 3 Models and formalization

### 3.1 DDoS attack

DDoS attacks, stemming from DoS attacks, utilize distributed attack networks to unleash extensive streams of assaults. Unlike DoS attacks targeting a single entity, DDoS attacks can concurrently employ various methods against multiple targets, exhibiting greater destructiveness and complexity, making tracking of the attackers challenging.

**Fig 1:**
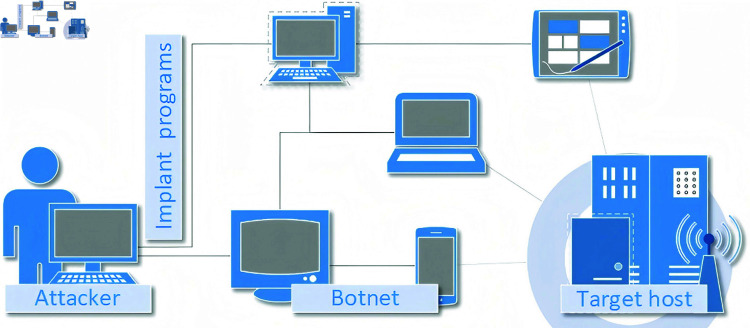
Illustration of a DDoS attack.

In the realm of DDoS attacks (Fig 1), assailants can emerge from any network host, controlling the entire assault process. By harnessing hundreds or thousands of controlled nodes, including main control consoles and proxy hosts, attackers coordinate extensive assaults to overwhelm designated targets. These attacks, involving resource-intensive activities like bandwidth, CPU, and memory consumption, aim to disrupt victim performance, potentially causing paralysis, system crashes, and denying access to legitimate users. Main control consoles and proxy hosts operate within a two-tiered structure, each running illicitly implanted attack programs. Main control consoles manage multiple proxy hosts and communicate commands received from the attacker to these hosts. Communication dynamics vary depending on the specific DDoS tool used; for example, Trinoo uses the UDP protocol, TFN uses ICMP via ICMP_ECHOREPLY packets, and stacheldraht employs TCP and ICMP protocols.

### 3.2 DDoS detection model

In the context of a zombie network targeting a victim’s server or network, each zombie host dispatches requests to the target’s IP address. This activity may result in an overwhelming load on the server or network, culminating in a denial of service for legitimate traffic. The challenge arises from the legitimate status of each zombie host as a standard internet device, making the distinction between attack traffic and normal traffic inherently complex. Currently, countering DDoS attacks remains a formidable challenge, with prevention taking precedence. Among various preventative measures, the deployment of firewalls at critical network nodes emerges as a prevalent and widely applicable strategy for DDoS attack prevention. This approach adeptly defends against or mitigates various common DDoS attacks, ensuring the uninterrupted operation of internal network hosts.

Next Generation Firewall (NGFW [17]), an influential and broadly applicable means of DDoS attack prevention, assumes a pivotal role in fortifying internal network hosts. NGFW, short for Next Generation Firewall, is engineered to comprehensively address threats at the application layer. Through meticulous analysis of network traffic and the application of traffic detection algorithms, NGFW devices adeptly identify various types of network attacks, implementing corresponding measures to shield internal networks from malicious activities.

**Fig 2:**
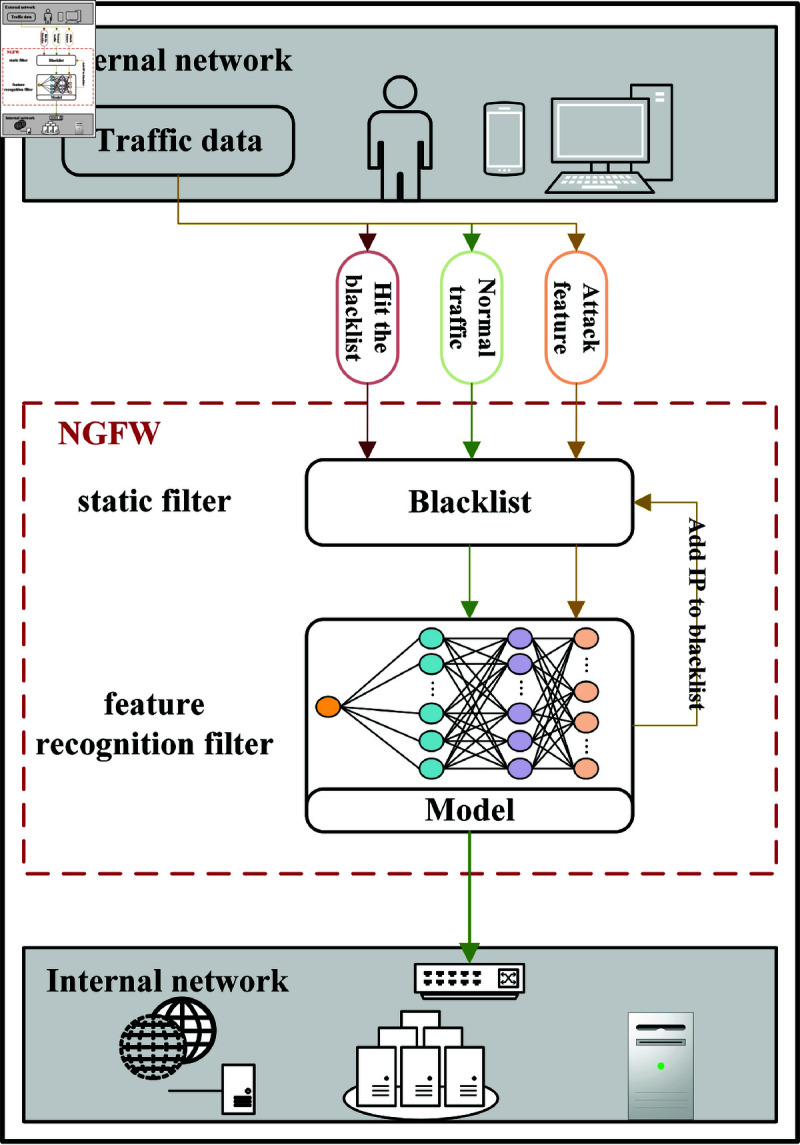
Illustration of a NGFW.

In Fig 2, NGFW devices conduct a thorough analysis of each traffic flow during runtime. Operating within a defense framework comprised of static filtering and feature recognition filtering, these devices effectively pinpoint various attack types, including traffic-based attacks and application-based attacks. This intricate defense mechanism ensures precise protection against a diverse range of DoS/DDoS attack flows. Static filtering involves the firewall’s swift action in discarding traffic from IP addresses listed in the blacklist or permitting traffic from IP addresses listed in the whitelist to pass through unhindered. Complementing this, feature recognition filtering relies on packet capture analysis to discern traffic characteristics. This method is instrumental in preventing attack flows initiated by zombie tools or through proxies, distinguishing them from the normal access behavior of legitimate users. Packet capture analysis entails the meticulous examination of packets within the traffic, extracting essential characteristics and employing feature recognition to determine the presence of malicious attack data in the current traffic packet. If new malicious attack flows are identified, the corresponding IP address is added to the blacklist for reference in static filtering. This comprehensive traffic filtering process underscores the firewall’s pivotal role as an effective and precise protective barrier.

### 3.3 Notations and problem formalizations

In the given exposition, it becomes apparent that the feature recognition filtering process necessitates meticulous traffic monitoring. The construction of the blacklist is contingent on the outcomes of feature recognition filtering. Thus, the crux of the entire procedure lies in the detection of malicious traffic.

This study utilizes a deep learning model to perform the task of detecting malicious traffic. The detection process can be mathematically expressed by the following equation:

$$y_{t}=f_{\theta }(X_{t})$$ (1)

Here, *f* denotes the deep neural network model parametrized by θ. *X*_*t*_ represents the model’s input at time *t*, sourced from the training set *D*_*train*_, while *y*_*t*_ signifies the actual output of the model at time *t*.

To optimize the model parameters, we define *L*(*y*,*Y*_*t*_) as the loss function at time *t*, which measures the difference between the model’s predicted output *y* and the true labels *Y*_*t*_. The objective during the parameter optimization process is to minimize the function:

$$J(\theta )=\frac{1}{\left|D_{train}\right|}\sum_{(X_{t},Y_{t})\in D_{train}}L(y_{t},Y_{t})$$ (2)

Consequently, the optimized parameters $\theta^{*}$ are determined as:

$$\theta^{*}=argmin_{\theta }J(\theta )$$ (3)

Finally, to assess the model’s efficacy, we introduce an evaluation metric $Eval(f_{\theta }(x_{t}))$, where *x*_*t*_ is drawn from the test set *D*_*test*_.

## 4 Methods

### 4.1 Overview

**Fig 3:**
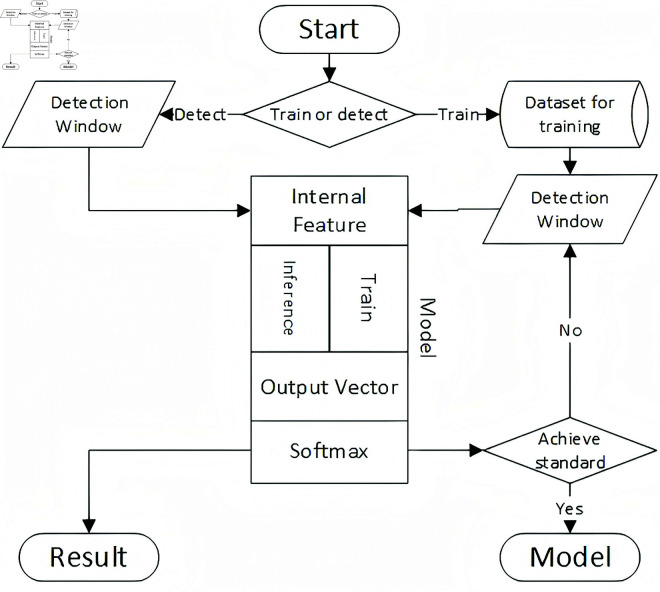
Algorithm process.

The proposed methodology in this manuscript centers on the processing of captured network traffic data. Internally, the model generates traffic samples for deep learning and constructs a network model based on Informer. From these traffic samples, the model extracts salient features for distinguishing between normal and abnormal traffic, as depicted in Fig 3. Ultimately, this process yields a model suitable for intrusion detection.

In contrast to manually designing features, feature extraction typically requires expertise prior to task execution. Moreover, conventional machine learning fails to provide guidance for optimizing this feature engineering, as there is no explicit method for improving optimization prompts for features. If the model’s performance is subpar, it becomes challenging to discern whether the issue lies with the features or the model.

However, the algorithmic model developed in this paper integrates the obtained feature vectors seamlessly with the tasks it performs. These features undergo continuous improvement during the training process through optimization algorithms. The model operates end-to-end, seamlessly transitioning from inputting network traffic data to executing the intrusion detection task.

### 4.2 Informer model

**Fig 4:**
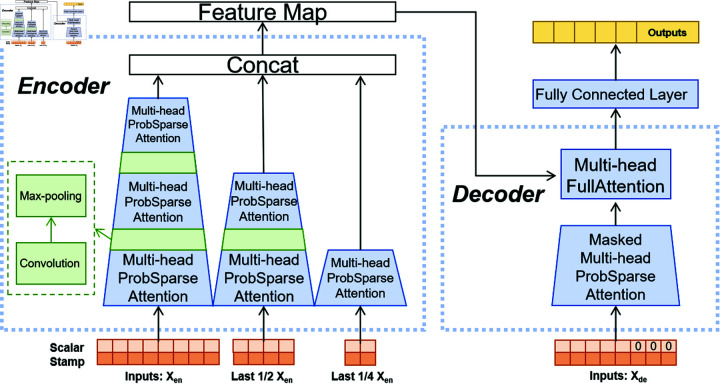
Structure of informer.

The “Informer” model is an innovative model developed by Haoyi Zhou and his team. The model addresses the limitations of the traditional Transformer model in handling LSTF tasks, such as those with high computational complexity and memory usage. Key innovations of the Informer model include the ProbSparse self-attention mechanism, which significantly reduces computational load; the self-attention distillation mechanism, which focuses on relevant data, and the generative-style decoder mechanism, which enhances forecasting accuracy. Much like the Transformer, the Informer model comprises an encoder and decoder, as shown in Fig 4 [16]. Nevertheless, what distinguishes the Informer model is its innovative approach.

The Informer model includes the following mechanisms:

- The ProbSparse self-attention mechanism, with a time complexity of O(L×log(L)), outperforms the standard attention mechanism, which has a time complexity of $\text{O}({\text{L}}^{2}$), where ’*L*’ represents the length of sequence.
- The self-attention distillation mechanism reduces the input sequence length at each layer, effectively lowering both computational and storage requirements.
- The generative decoder mechanism, it yields results in a single step during sequence prediction, in contrast to the step-by-step approach. This directly reduces the prediction time complexity from *O*(*N*) in Transformer to *O*(1).

**Fig 5:**
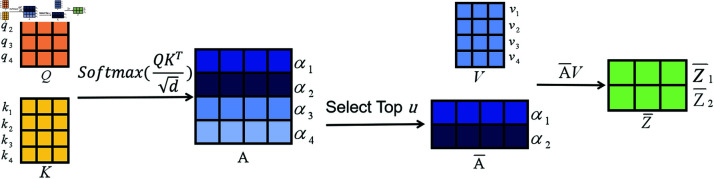
ProbSparse self-attention mechanism.

In traditional self-attention mechanisms, it has been observed that some rows in the attention score matrix exhibit probability distributions that are close to uniform, suggesting that only a subset of the query-key dot products contribute to the attention mechanism. As illustrated in Fig 5, the top *u* queries with the most significant contributions are selected, and their corresponding attention scores form a new attention score matrix $\overset{―}{A}$. This matrix is then multiplied by the matrix *V* to obtain the feature extraction $\overset{―}{Z}$. After averaging the *v* matrix, it is inserted into the corresponding positions in $\overset{―}{Z}$ that were removed, resulting in the output *Z* of the sparse attention mechanism.The most contributive queries discriminants are obtained from the equation:

$$M(q_{i},K)=\ln\sum_{j=1}^{L_{K}}e^{\frac{q^{i}k_{j}^{T}}{\sqrt{d}}}-\frac{1}{L_{K}}\sum_{j=1}^{L_{K}}\frac{q^{i}k_{j}^{T}}{\sqrt{d}}$$ (4)

Where *q*_*i*_, *k*_*i*_ stand for the *i*-th row in *Q*, *K*. *L*_*K*_ represents the number of rows of K.We can determine the corresponding *u* queries by selecting the top *u* values of *M*(*q*_*i*_,*K*), thereby implementing the coefficient attention mechanism.

In the Encoder, Informer incorporates multiple stacks. Each stack takes a vector representation of tokens as input, which is then subjected to convolution through a Conv1d layer (applied along the sequence). This operation produces a representation of size $L\;\times \;d_{model}$, where *d*_*model*_ signifies the dimensionality of the token vectors. Subsequently, these two representations are combined to create the input for the Attention Block. This representation then traverses through multiple Attention Blocks, each featuring multi-head ProbSparse Self-Attention. The output from each block subsequently undergoes a Conv1d convolutional layer, an ELU activation layer, and a Maxpooling layer with a stride of 2. The specific formulas are as follows:

$$X_{t}^{j+1}=MaxPool(ELU(Conv1d(X_{t}^{j})))$$ (5)

In this context, $X_{t}^{j}$ denotes the input to the current layer, while $X_{t}^{j+1}$ represents the output from the same layer. This iterative process results in the reduction of the representation size from the initial block to *L*/2. Following a series of sequential operations, the ultimate downsized representation, referred to as the Feature Map, is achieved.

The Decoder employed by Informer bears resemblance to traditional counterparts. To facilitate the generation of extended output sequences, Informer necessitates the following input structure:

$$X_{t}^{feed\_de}=Concat(X_{t}^{token},X_{t}^{o})\in R^{(L_{token}+L_{y})\times d_{model}}$$ (6)

The input is bifurcated into two segments: the initial half encompasses the start token sequence, while the latter half represents the predictive target. Scalar values are padded with zeros, and timestamps adhere to the definition provided earlier. Subsequently, the sequence traverses a Masked ProbSparse Self-Attention layer and ultimately undergoes a fully connected layer to derive the final output.

### 4.3 Informer-based DDoS attack detection method

In the preceding explanation, each detection input corresponds to a traffic window. A window comprises L traffic data entries, and the dimensionality of each input vector for the data is represented by *d*_*input*_. Thus, a single input is a two-dimensional tensor with dimensions $L\;\times \;d_{input}$, After embedding, the dimensionality of the tensor is changed to $L\;\times \;d_{model}$. Each probsparse Self-Attention consists of *h* Attention heads, and each head produces an output with dimension *n*_*h*_. Consequently, the output dimension of a probsparse Self-Attention is $L\times (n_{h}*h)$.

**Fig 6:**
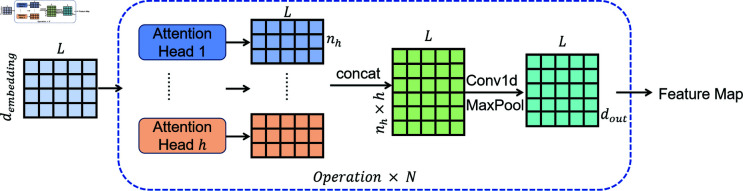
Structure of encoder.

Since this study does not focus on predictive tasks, only the Encoder is employed for traffic feature extraction. To adapt to the feature extraction task, in the previously introduced Informer structure, each attention, 1D convolution and max pooling operation is applied to the feature dimension rather than the temporal dimension. The Encoder structure of the model used in this study is shown in Fig 6.

In the Decoder stage, fully connected layers and a Softmax function are used for classification. By linking the output of the Encoder to a fully connected layer and then applying Softmax, the detection results for the initial input vector, with dimensions L×1, are directly produced. At this point, the detection results for each traffic entry within the input traffic window are obtained.

## 5 Experiment

### 5.1 Experimental environment

The experiments were conducted on a Linux server equipped with four NVIDIA A100 80GB GPUs, and powered by an Intel Xeon Gold 6248 CPU, 128 GB of RAM, and a 100 TB SSD to ensure fast data access and efficient storage. The operating system used is Ubuntu 20.04, and the software stack includes Python 3.11, along with PyTorch 2.0 for model development and training. Data manipulation and analysis are performed using Pandas 2.0.3 and NumPy 1.26.4.

### 5.2 Dataset and preprocessing

This study utilizes the CICIDS-2018 dataset, created by the Canadian Communication Security Establishment and the Canadian Institute for Cybersecurity, featuring network traffic data from real-world DDoS scenarios. The dataset comprises 80 features, 1 label, and labeled instances of both normal and attack traffic.

Specifically, traffic data from two days—Wednesday-21-02-2018 and Friday-02-03-2018—were selected, covering normal traffic and three attack types: Bot, DDoS-LOIC-UDP, and DDoS-HOIC. LOIC (Low Orbit Ion Cannon) refers to a specific type of DDoS attack. The network traffic undergoes segmentation, and the distribution of anomaly traffic is detailed in Table 1.

**Table 1:** Distribution of traffic categories.

Sample Type	Friday	Wednesday
Benign	762 384	360 833
Bot	286 191	0
LOIC-UDP	0	1 730
HOIC	0	686 012
Total	1 048 975	1 048 575

The dataset was first cleaned by removing entries with missing values and duplicates. To minimize reliance on expert knowledge and enhance adaptability, an end-to-end approach was used for DDoS attack detection, enabling the model to learn features directly from raw data. All original features were retained, and timestamps were transformed into numerical features, resulting in a feature dimension (*d*_*input*_) of 84. To address class imbalance, random undersampling was applied to overrepresented classes and oversampling to underrepresented ones (e.g., HOIC), ensuring a balanced sample distribution.

Then uniformly sampled 100,000 data windows, each with a window size *L* of 96, from the entire dataset as model inputs. The windows were randomly shuffled, with 70,000 allocated to the training set and 30,000 to the test set. Subsequently, Z-score normalization was independently applied to the training and test sets, resulting in the finalized dataset.

### 5.3 Experimental details

In this experiment *d*_*model*_ = 512. The Encoder of the Informer model consists a single 3-layers of sparse attention stack(Dropout = 0.1), and each attention operation is followed by a Conv1D and a MaxPool1D operation. For the first layer of ProbSparse self-attention mechanism, we let the number of attention heads *h*_1_ is 4, and each head produces an output with dimension *n*_*h*1_ = 128. Subsequently, each 1D convolution uses a kernel size of 3×2, and the Max pooling stride is set to 2. Use the ELU activation function between the convolution and pooling layers. For the second layer *h*_2_ is 4, and each head produces an output with dimension *n*_*h*2_ = 64. For the third layer *h*_3_ is 4, and each head produces an output with dimension *n*_*h*2_ = 32. As a result, the final output dimension of the Encoder is 96×64. The Decoder part directly uses two fully connected layers, with 512 neurons in the hidden layer, GELU as the activation function, and a dropout rate of 0.1. Finally, a softmax classifier is connected to produce a classification output with dimensions of 96×1.

For training, We used cross-entropy loss to evaluate detection performance, with lower values indicating better accuracy. The Adam optimizer, with an initial learning rate of 0.0001, was employed, alongside a learning rate decay strategy that halved the rate at the end of each epoch. The model was trained for 6 epochs with a batch size of 32. Training stopped when the loss difference between consecutive epochs dropped below 0.001, indicating convergence.

### 5.4 Results

For the assessment of model performance, this paper employs the confusion matrix calculation. Table 2 represents a binary classification confusion matrix. The extension of the confusion matrix to multiclass can be achieved by considering one class as the positive class and the rest as negative classes.

**Table 2:** Confusion matrix.

Type	Predicted as true	Predicted as false
Actual is true	TP	FN
Actual is false	FP	TN

Accuracy (*acc*): The ratio of correctly classified samples by the model, often referred to as accuracy, is defined by the following formula:

$$acc=\frac{TP+TN}{TP+TN+FP+FN}$$ (7)

Precision (*p*): The proportion of samples predicted as class C among those that are actually of class C, as defined by the formula:

$$p=\frac{TP}{TP+FP}$$ (8)

Recall (*r*): The proportion of samples with the actual class C among those predicted as class C by the model, as defined by the formula:

$$r=\frac{TP}{TP+FN}$$ (9)

F1 Score (*F*1–*score*): The F1 score, calculated as the harmonic mean of precision (p) and recall (r), offers a balanced measure by combining both metrics. A higher F1 score signifies improved model performance. It is defined by the following formula:

$$F1-score=2\times \frac{p\times r}{p+r}$$ (10)

Accuracy, precision, recall, and F1 score were selected to provide a comprehensive evaluation of model performance. Accuracy measures overall prediction correctness, but may be insufficient for imbalanced datasets. Precision focuses on the correctness of positive predictions, while recall assesses their completeness. The F1 score, as the harmonic mean of precision and recall, balances both metrics, offering a more robust evaluation when false positives and false negatives are critical. This combination ensures a thorough and balanced assessment of the model’s effectiveness.

**Fig 7:**
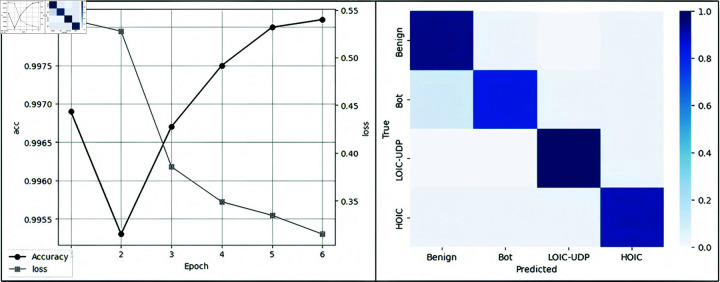
Model performance.

The proposed model demonstrated outstanding performance in experiments, achieving a final accuracy of 99.81% as shown in Fig 7, highlighting its strong generalization capability. With a precision of 99.95%, the model effectively minimizes false positives, ensuring that legitimate traffic is not misclassified as malicious. The recall rate of 99.56% indicates its high effectiveness in detecting nearly all DDoS attack instances, reducing the risk of undetected threats. Additionally, the F1 score of 99.75% reflects a well-balanced trade-off between precision and recall. The stable learning curve throughout training, without signs of overfitting, further confirms the model’s robustness and reliability. Overall, these results demonstrate the model’s exceptional ability to detect DDoS attacks in complex and dynamic network environments in real-time.

**Fig 8:**
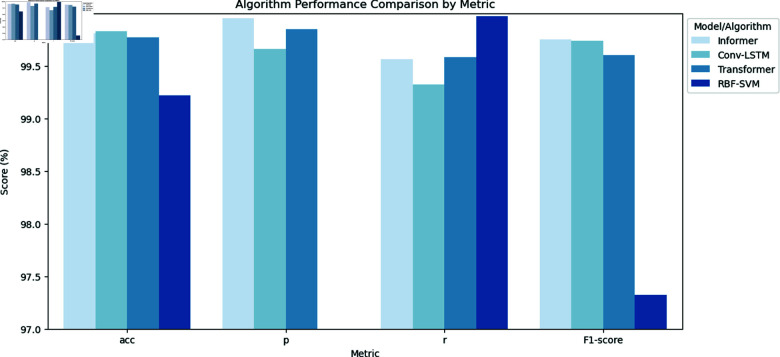
Algorithm comparison.

Simultaneously, this study conducted tests on alternative deep learning algorithms, including Conv-LSTM, Transformer, and RBF-SVM. The results, presented in Table 3 and Fig 8, highlight that Informer indeed exhibits a notable advantage in the detection of DDoS attack traffic.

**Table 3:** Algorithm comparison.

Model/Algorithm	acc	p	r	F1-score
Informer	99.81%	99.95%	99.56%	99.75%
Conv-LSTM	99.83%	99.66%	99.32%	99.74%
Transformer	99.77%	99.85%	99.58%	99.60%
RBF-SVM	99.22%	94.81%	99.97%	97.32%

To evaluate the stability and reliability of the proposed Informer DDoS detection model, this study employs the Bootstrap resampling method to statistically analyze key performance metrics. The experimental procedure was designed to assess the stability of the model’s performance metrics through Bootstrap resampling. Specifically, the resampling process was conducted 100 times. In each iteration, 100 data windows were randomly sampled with replacement from the original test set. This approach ensures that each resampled dataset maintains the same structure as the original data while introducing variability for statistical analysis. For every resampled dataset, the model’s Accuracy, Precision, Recall, and F1-score were calculated. Finally, the distribution of these metrics was analyzed to estimate their means and corresponding 95% confidence intervals, providing insights into the model’s robustness and generalizability. The performance of the model under Bootstrap resampling is summarized in the following Table 4

**Table 4:** Performance Metrics with 95% Confidence Intervals (Bootstrap Resampling, *B* = 100).

Metric	Mean	Standard Deviation	95% Confidence Interval (CI)
Accuracy	0.9979	0.0032	[0.9916, 1.0000]
Precision	0.9996	0.0025	[0.9947, 1.0000]
Recall	0.9961	0.0041	[0.9881, 1.0000]
F1-score	0.9977	0.0031	[0.9916, 1.0000]

## 6 Conclusion and future work

This study successfully implements an end-to-end DDoS attack detection framework based on the Informer model. A notable advantage of this approach is its ability to eliminate the need for manual feature extraction by directly constructing real-time representations from raw traffic data. Its key strength lies in its capacity for real-time detection, as evidenced by experimental results that demonstrate superior performance compared to several existing detection algorithms.

Despite its effectiveness, certain aspects of the proposed method warrant further refinement. For instance, the full predictive potential of the Informer model remains underexplored, as the current approach exclusively utilizes the Encoder for direct traffic classification. Additionally, the classification scheme is limited to benign and attack categories, overlooking the detailed taxonomy of attack types available in the CICIDS-2018 dataset. These limitations highlight the need for further enhancement.

Future research should consider the distributed nature of IoT devices, leveraging their collective detection capabilities. Investigating joint detection strategies across distributed devices could significantly improve the reliability and generalizability of DDoS attack detection in complex network environments.

## References

[1] Fox J, Donnellan A, Doumen L. The deployment of an IoT network infrastructure, as a localised regional service. 2019 IEEE 5th World Forum on Internet of Things (WF-IoT). 2019. p. 319–24. doi: 10.1109/wf-iot.2019.8767188

[2] Gupta AK, Johari R. IOT based electrical device surveillance and control system. In: 2019 4th International Conference on Internet of Things: Smart Innovation and Usages (IoT-SIU). 2019. p. 1–5.

[3] Hao Y, Duan Z, Yao T, Zhang T, Huo R. Study on reliability system of a monitoring device for power transmission and transformation based on transportation and inspection IOT. In: 2022 European Conference on Communication Systems (ECCS). 2022. p. 43–7. doi: 10.1109/eccs54035.2022.00017

[4] Yu KG, Lin XH, Cheng XM, Xiao XQ, Zhan B, Yu L, Liu YL, Xu Q. Heterogeneous IoT and data fusion communication algorithms for power distribution station areas. In: 2022 3rd International Conference on Computer Vision, Image and Deep Learning & International Conference on Computer Engineering and Applications (CVIDL & ICCEA). 2022. p. 1011–5.

[5] Spehar J, Fuks A, Vauclair M, Meijer M, van Beek J, Shao B. Power challenges caused by IOT edge nodes: securing and sensing our world. In:2019 31st International Symposium on Power Semiconductor Devices and ICs (ISPSD). 2019. p. 17–22.

[6] Rohit MH, Fahim SMd, Khan AHA. Mitigating and detecting DDoS attack on IoT environment. In: 2019 IEEE International Conference on Robotics, Automation, Artificial Intelligence and Internet of Things (RAAICON). 2019. p. 5–8.

[7] Atmadja AR, Purwarianti A. Comparison on the rule based method and statistical based method on emotion classification for Indonesian Twitter text. In: 2015 International Conference on Information Technology Systems and Innovation (ICITSI). 2015. p. 1–6. doi: 10.1109/icitsi.2015.7437692

[8] Feinstein L, Schnackenberg D, Balupari R, Kindred D. Statistical approaches to DDoS attack detection and response. In: DARPA Information Survivability Conference and Exposition. 2003. p. 303–14.

[9] Prasad KM, Reddy DAR, Rao KVG. DoS and DDoS attacks: defense, detection and traceback mechanisms - a survey. Glob J Comput Sci Technol. 2014;14:15–32.

[10] Dong B, Wang X. Comparison deep learning method to traditional methods using for network intrusion detection. In: 2016 8th IEEE International Conference on Communication Software and Networks (ICCSN). 2016. p. 581–5. doi: 10.1109/iccsn.2016.7586590

[11] Kimanzi R, Kimanga P, Cherori D, Gikunda PK. Deep learning algorithms used in intrusion detection systems – a review. In: 2024 IEEE International Conference on Power, Electrical, Electronics and Industrial Applications (PEEIACON). 2021;101574-99.

[12] Saheed YK, Abdulganiyu OH, Tchakoucht TA. Modified genetic algorithm and fine-tuned long short-term memory network for intrusion detection in the internet of things networks with edge capabilities. Appl Soft Comput. 2024;155:111434. doi: 10.1016/j.asoc.2024.111434

[13] Saheed YK, Omole AI, Sabit MO. GA-mADAM-IIoT: a new lightweight threats detection in the industrial IoT via genetic algorithm with attention mechanism and LSTM on multivariate time series sensor data. Sens Int. 2025;6:100297. doi: 10.1016/j.sintl.2024.100297

[14] Vaswani A, Shazeer N, Parmar N, Uszkoreit J, Jones L, Gomez AN, Kaiser L, Polosukhin I. Attention is all you need. In: 31st International Conference on Neural Information Processing Systems (NIPS’17). 2017. p. 6000–10.

[15] Ahmed SW, Kientz F, Kashef R. A Modified Transformer Neural Network (MTNN) for robust intrusion detection in IoT networks. In: 2023 International Telecommunications Conference (ITC-Egypt). 2023. p. 663–8.

[16] Zhou H, Zhang S, Peng J, Zhang S, Li J, Xiong H, Zhang W. Informer: beyond efficient transformer for long sequence time-series forecasting. In: AAAI Conference on Artificial Intelligence. 2020. p. 402.

[17] Neupane K, Haddad R, Chen L. Next generation firewall for network security: a survey. SoutheastCon 2018. 2018. p. 1–6.

